# M. Lebert on Cancer

**Published:** 1853-04

**Authors:** 


M. Lebert *on Cancer.*—M. Lebert, the author of the well-known work on Cancer, lately read a paper before the Surgical Society of Paris, on Fibro-plastic Tumors. The principal facts and conclusions brought forward by M. Lebert are the following:—

1. Fibro-plastic tumors are composed of a cellular texture of new formation, similar to that seen in embryonic life. Fibro-plastic tissue may have an inflammatory origin, it may be the result of hypertrophy, or arise quite spontaneously.

2. Fibro-plastic tumors are made up of a soft lobulated tissue, of a pinkish yellow; or else of a firmer texture, of a pale hue, and more homogenous; or, finally, of a mixture of a gelatiniform substance with the two structures just mentioned. These tumors are situated in the skin, or the subcutaneous areolar tissue; they may be buried in the depth of the muscular masses of a limb, especially the thigh; or else they may spring up in bone, a favorite seat being the jaws; or finally, they may grow upon the meninges.

3. Fibro-plastic tumors present the same characters in man and in domestic animals.

4. The Fibro-plastic texture, which owes its origin to inflammation, has a marked tendency to fibrous transformation; it is often met with in protracted inflammations, of which it is at the same time the support and the produce.

5. Fibro-plastic tumors present either a smooth or lobulated surface, and are either globular or flattened, according to their seat, and in cases of hypertrophy they assume the shape of the affected organ. They are generally free and well-defined; but when springing from bone they are sessile, and look like excrescences.

6. These tumors may become very large, both in glandular organs and when seated on limbs, especially when they contain much gelatiniform tissue. They are generally surrounded by a fibro-cellular envelope. When a section is made in a recent case the liquid obtained is clear and transparent; but it is sometimes thick and dull, when flocculi or entire lobuli are suspended in it. Besides the textures above mentioned, there is sometimes found in the tumors a substance of pale yellow, and quite dull; sometimes calcareous concretions are found, or else osseous radiations, when the disease begun in the periosteum.

7. Under the microscope, cells with small, rounded, or ovoid nuclei are seen, and all the forms intermediate between the cell and common fibre, especially a great many narrow fusiform bodies; the nucleoli are always very small. There are also perceived original cells with compound nuclei rolled up together, and sometimes fusiform nuclei which then constitute fibro-plastic masses.

8. As regards neighboring textures, fibro-plastic tumors grow like fibrous tumors, as they press upon these textures without taking their place; whilst cancerous tumors substitute themselves in neighboring parts. Fibro-plastic tumors follow, as to their growth, the following development:—There is first a local deposit; the increase is then slow, but soon becomes rapid; phlegmasia takes place; then comes hæmorrhage, partial calcification, softening, the formation of cysts, partial ossification, ulceration, and then, in exceptional cases, sloughing.

9. Both sexes are obnoxious to these tumors; they are seen at all ages, but they arise more frequently between thirty-five and fifty years.

10. Recurrence after operation takes place, especially in autogeneous tumors, and has not been observed in fibro-plastic hypertrophy; recurrence is not observed, as in cancer, to arise in distant organs, though it is often caused by an incomplete operation. Notwithstanding the occasional pertinacity of fibro-plastic tumors to recur in the cicatrix, they are not found to injure the general health. In very rare cases a kind of fibro-plastic diathesis becomes established; this fact has, however, been observed with fibrous, adipose, neuromatous, and melanotic tumors.

11. Singleness and a strictly local nature have been observed as a rule in fibro-plastic tumors. This fact is proved by twenty-six carefully conducted autopsies of cases in which the disease had reached its natural termination, and also by a considerable number of perfect and permanent cures after operation; whilst no such cures are known to exist respecting true cancer.

12. Fibro-plastic tumors have a much more chronic character than cancerous tumors; and save a few cases of very rapid growth, it may be said that fibro-plastic tumors, when they become fatal, last rather more than between five and ten years; whilst the natural duration of cancer is from two to two years and a half. Cases have even been known, and are not rare, in which fibro-plastic tumors have lasted twenty years and more, without much inconvenience. It should, however, be mentioned that these tumors, after a very slow progress, have rapidly assumed a very serious character.

13. Pain is very rarely acute and permanent, and is mostly, as in other tumors, of a neuralgic kind. Functional disturbance depends principally on the seat of the tumor. The general health, in the great majority of cases, remains undisturbed, except in the very rare cases of general fibro-plastic infection, or those in which the local affection has ended in extensive ulceration.

14. The prognosis of fibro-plastic tumors is much more favorable than that of cancer. Curability is the rule. The fibro-plastic texture of inflammatory origin is the more likely to be benign the more it shows a tendency to assume a fibrous character. The prognosis is, in fibro-plastic hypertrophy, likewise favorable. In fact these tumors constitute a local affection little or not at all likely to recur, which can only become dangerous in so far as its seat, extent, or amount of ulceration are concerned. Kelis is likewise an affection of a simple kind, (see "Mirror of Hospitals," The Lancet, vol. ii., 1852, p. 567,) notwithstanding its pertinacity regarding recurrence in the cicatrix after operation. Fibroplastic tumors of spontaneous origin are very likely to recur after excision, when they are situated on the tegumentary surface, under the skin, or in the bones. The surgeon should always bear in mind the possibility of general infection, only, however, as an occasional occurrence, but not as a probability. Fibro-plastic tumors seated in the meninges destroy the patient by interfering with the functions of the brain, though remaining all the while a perfectly local affection.

15. The treatment of these tumors should begin with the prolonged use of preparations of iodine, when the origin is of a syphilitic nature, or when the tumor is simply owing to hypertrophy. When an operation is necessary, it should consist, as a rule, of complete extirpation; strong caustics should be tried with superficial tumors of small dimension. Operations in such cases should always be performed boldly, whether the knife be carried into soft parts, or whether the affection be seated in bone. Amputation should be performed when the tumor is situate in the depth of the limb and the former sends prolongations into parts where dissection is difficult.

Autoplasty should be used to substitute healthy tissue to cicatrices likely to favor recurrence, in operations of a moderate extent,and when the tumor is seated on the surface. The actual cautery is necessary when superficial tumors are too extensive to admit of autoplasty.

It is finally advisable to use iodine *after* operations, as a prophylactic means against recurrence of general infection.—*London Lancet.*

